# Why Can’t Rodents Vomit? A Comparative Behavioral, Anatomical, and Physiological Study

**DOI:** 10.1371/journal.pone.0060537

**Published:** 2013-04-10

**Authors:** Charles C. Horn, Bruce A. Kimball, Hong Wang, James Kaus, Samuel Dienel, Allysa Nagy, Gordon R. Gathright, Bill J. Yates, Paul L. R. Andrews

**Affiliations:** 1 Biobehavioral Medicine in Oncology Program, University of Pittsburgh Cancer Institute, Pittsburgh, Pennsylvania, United States of America; 2 Department of Medicine: Division of Gastroenterology, Hepatology, and Nutrition, University of Pittsburgh School of Medicine, Pittsburgh, Pennsylvania, United States of America; 3 Department of Anesthesiology, University of Pittsburgh School of Medicine, Pittsburgh, Pennsylvania, United States of America; 4 Center for Neuroscience, University of Pittsburgh, Pittsburgh, Pennsylvania, United States of America; 5 United States Department of Agriculture, Animal and Plant Health Inspection Service, Wildlife Services, National Wildlife Research Center, Monell Chemical Senses Center, Philadelphia, Pennsylvania, United States of America; 6 Department of Biostatistics, University of Pittsburgh, Pittsburgh, Pennsylvania, United States of America; 7 Department of Neuroscience, University of Pittsburgh, Pittsburgh, Pennsylvania, United States of America; 8 United States Department of Agriculture, Animal and Plant Health Inspection Service, Wildlife Services, National Wildlife Research Center, Fort Collins, Colorado, United States of America; 9 Department of Otolaryngology, University of Pittsburgh School of Medicine, Pittsburgh, Pennsylvania, United States of America; 10 Division of Biomedical Sciences, St. George’s University of London, London, United Kingdom; INRA, France

## Abstract

The vomiting (emetic) reflex is documented in numerous mammalian species, including primates and carnivores, yet laboratory rats and mice appear to lack this response. It is unclear whether these rodents do not vomit because of anatomical constraints (e.g., a relatively long abdominal esophagus) or lack of key neural circuits. Moreover, it is unknown whether laboratory rodents are representative of Rodentia with regards to this reflex. Here we conducted behavioral testing of members of all three major groups of Rodentia; mouse-related (rat, mouse, vole, beaver), Ctenohystrica (guinea pig, nutria), and squirrel-related (mountain beaver) species. Prototypical emetic agents, apomorphine (sc), veratrine (sc), and copper sulfate (ig), failed to produce either retching or vomiting in these species (although other behavioral effects, e.g., locomotion, were noted). These rodents also had anatomical constraints, which could limit the efficiency of vomiting should it be attempted, including reduced muscularity of the diaphragm and stomach geometry that is not well structured for moving contents towards the esophagus compared to species that can vomit (cat, ferret, and musk shrew). Lastly, an *in situ* brainstem preparation was used to make sensitive measures of mouth, esophagus, and shoulder muscular movements, and phrenic nerve activity–key features of emetic episodes. Laboratory mice and rats failed to display any of the common coordinated actions of these indices after typical emetic stimulation (resiniferatoxin and vagal afferent stimulation) compared to musk shrews. Overall the results suggest that the inability to vomit is a general property of Rodentia and that an absent brainstem neurological component is the most likely cause. The implications of these findings for the utility of rodents as models in the area of emesis research are discussed.

## Introduction

**Figure 1:**
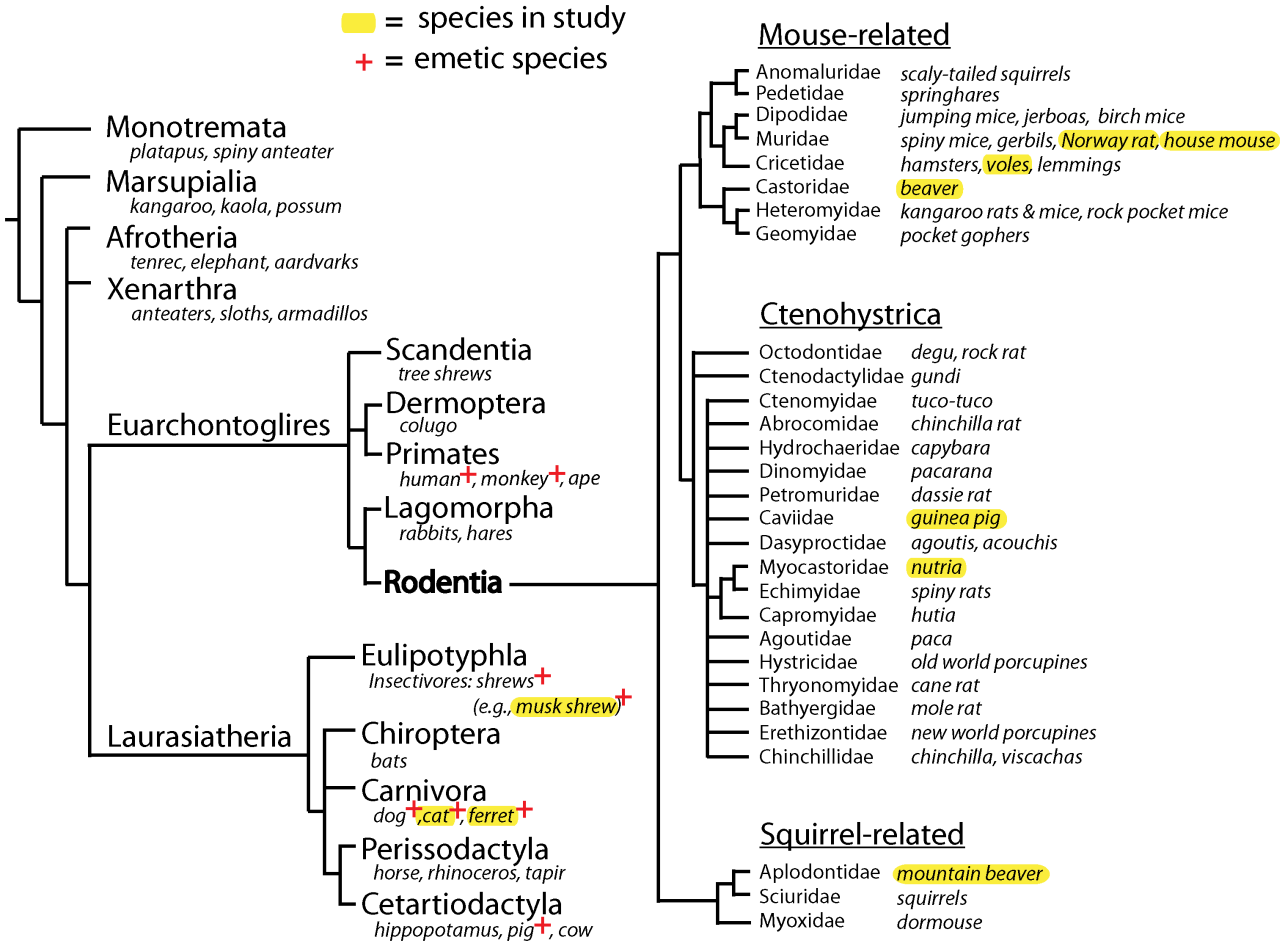
Mammalian phylogenetic tree [12], [18], [19]. Specific species listed in the tree branches are examples and may not include all those contained in each class; species included in the current study are marked with a yellow highlight. A “+” sign notes a species with a well established emetic response (demonstrated in laboratory studies) (e.g., [10], [11], [23], [42], [50], [76]–[79]).

The presence of the vomiting (emetic) reflex is widespread among mammals. Members of several major lineages, including carnivores (e.g., cat, dog, ferret [1]–[6]), primates (e.g, human, monkey [7], [8]), and insectivores (e.g., shrews [9]–[11]) possess this response (Fig. 1). The vomiting reflex is reportedly not present in Rodentia, ∼40% of all mammalian species (and Lagomorpha – rabbits and hares) [12], but there has been limited study with most reports focused on species of common laboratory rodents (i.e., derivatives of Norway rats and house mice). Because of the lack of the vomiting reflex in laboratory rats and mice it has been problematic to study responses to emetic agents in these species and, therefore, other behavioral markers have been used extensively, such as conditioned taste aversion and pica testing (clay ingestion) (see review [13]). However, two significant questions remain essentially unanswered with regard to a lack of the vomiting reflex in laboratory rodents: 1) are laboratory rats and mice representative of other rodents?, and 2) what is the cause of the inability to vomit in these species? The lack of emetic responses has been attributed to differences in upper alimentary tract anatomy and neural circuitry [14], [15] but these hypotheses have not been extensively tested. Understanding the lack of emesis in rodents has implications for the suitability of typical laboratory species, such as rats and mice, for the study of nausea and vomiting (Chap. 8 in [16]; [17]).

The current study focused on directly addressing these questions by conducting emetic testing and making anatomical measurements in species from the three major lineages of Rodentia: 1) Mouse-related (rat, mouse, vole, beaver), 2) Ctenohystrica (guinea pig, nutria), and 3) Squirrel-related (mountain beaver) species (Fig. 1) [18], [19]; and, comparing these results to species with a vomiting reflex (cat, ferret, and musk shrew) [1], [9], [20]. We used prototypical emetic agents, apomorphine (sc; a dopamine D_2_ receptor agonist), veratrine (sc; a plant alkaloid), and copper sulfate (ig; a gastric irritant), which are thought to produce emesis by acting on the area postrema [21], nodose ganglia [22], and abdominal vagal afferent fibers [8], respectively. These agents were also selected because they have been extensively used in emesis testing in a variety of species [5], [9], [21], [23]–[26]. We also measured anatomical parameters of the esophagus, diaphragm, and stomach of these species because it has been postulated that differences in these structures might constrain the ability of animals to vomit [14]. Comparison anatomical measures from species with an emetic response, shrews, ferrets, and cats, were included. Lastly, to determine if laboratory rodents possess subtle emetic-like responses (i.e., those that cannot be delineated in free moving observational studies) and to assess potential central motor patterning consistent with an emetic episode (i.e., multiple closely spaced retches [27]), we recorded mouth and esophageal movements using sensitive force transducers in an isolated *in situ* brainstem preparation of rats, mice, and musk shrews [28]–[30].

## Materials and Methods

All experiments were approved by Institutional Animal Care and Use Committees of the Monell Chemical Senses Center and University of Pittsburgh. Field station studies using beaver, mountain beaver, nutria, and vole were approved by the National Wildlife Research Center (United States Department of Agriculture, USDA).

### Animals

The species studied and demographics are listed in Table 1. Beaver (8 females), mountain beaver (sewellel; 6 females and 2 male), nutria (coypu; 9 males), Townsend’s vole (5 females and 4 males) were acquired from the USDA and tested at the USDA/APHIS/WS (USDA/Animal and Plant Health Inspection Service/Wildlife Services) National Wildlife Research Center in Olympia, WA, USA. Musk shrews (19 females and 3 males) were offspring from a colony maintained at the University of Pittsburgh Cancer Institute (a Taiwanese strain derived from stock supplied by the Chinese University of Hong Kong). Hartley guinea pigs (2 females and 2 males) were obtained from Charles River Laboratories (Wilmington, MA). Domestic short hair cats (8 males), Fitch ferrets (8 males), C57BL/6 mice (3 females and 14 males), and Sprague Dawley rats (26 males) were purchased from commercial vendors (Liberty Research, Waverly, NY, USA; Marshall BioResources, North Rose, NY, USA; Charles River, Kingston, NY, USA). Cats and ferrets were used *post mortem* in these anatomical studies and also used in other published emesis behavioral and physiology experiments [31], [32].

**Table 1:** Animals.

			Behavioral testing- n						In situ brainstem testing- n	
	Total n	Body weight mean ±SEM	Saline (sc)	Saline (ig)	Apomorphine (sc)	Veratrine (sc)	CuSO_4_ (ig)	Anatomy Measures- n	RTX	Vagal afterent stimulation
**Rodentia**										
**Mus musculus** (House mouse)	17	20±2 g	–	–	–	–	–	8	5	4
**Microtus townsendii** (Townsend’s vole)	9	92±22 g	2	2	2	3	2	4	–	–
**Rattus norvegicus** (Norway rat)	26	417±32 g 69±21 g*	4	4	4	4	4	4	6*	4*
**Cavia porcellus** (Guinea pig)	4	1.2±0.07 kg	2	–	4	4	3	4	–	–
**Aplodontia rufa** (Mt. Beaver)	8	1.3±0.1 kg	2	2	4	4	2	4	–	–
**Myocastor coypus** (Nutria)	9	6.5±0.9 kg 0.8±0.2 kg*	2	2	2 & 2*	2 & 2*	1 & 1*	5	–	–
**Castor Canadensis** (Beaver)	8	14.9±1.1 kg	2	2	1	3	2	4	–	–
**Emetic species**										
**Suncus murinus** (Musk shrew)	22	46±1.3 g	–	–	–	–	–	9	7	6
**Mustela putorius furo** (Ferret)	8	1.8±0.0 kg	–	–	–	–	–	8	–	–
**Felis catus** (House cat)	8	3.6±0.2 kg	–	–	–	–	–	8	–	–

* = juveniles.

All animals had *ad libitum* access to food and water except during test periods. Standard laboratory housing (plastic box with a top metal screen), water bottles, food, and lighting (12 h light/12 h dark) were used for mice (cage length×width×height, 28×17×12 cm; Lab diet 5015, PMI Nutrition International, St. Louis, MO), rats (cage length×width×height, 39×28×19 cm; Lab diet 5012, PMI Nutrition), musk shrews (cage length×width×height, 28×17×12 cm; mixture of 75% Purina Cat Chow Complete Formula and 25% Complete Gro-Fur mink food pellets, Milk Specialty, New Holstein, WI, USA), and voles (cage length×width×height, 20×31×13 cm; Lab diet 5012, PMI Nutrition). Guinea pigs (cage length×width×height, 49×55×29 cm; Guinea Pig Chow 5025, Dyets Inc., Bethelehem, PA, USA), ferrets (cage length×width×height, 61×64×43; Mazuri Ferret Diet, PMI Nutrition, St. Louis, MO, USA), and cats (cage length×width×height, 91×91×76 cm, Purina 5003 feline diet) were kept in stainless steel metal caging units with water bottles and cups or dispensers containing pelleted food. Larger species were kept in outdoor holding facilities (July to March; pen length×width×height, 3×5×1.5 m; Lab Diet 5012, PMI Nutrition, and diet enrichment consisting of apples, carrots, and dried corn on the cob; fresh water and pelleted rations were provided in stainless steel bowls) at the Wildlife Research Center in Olympia, WA, USA, including mountain beaver, nutria, and beaver. Nutria and beaver pens contained a 1000 L steel water tank and mountain beavers had an insulated den box made from a wooden log. All animals except mice (1 to 3 per cage) and rats (1 to 2 per cage) were singly housed. All behavioral testing was conducted during the light phase (0800–1700 h).

### Emetic Testing in Free Moving Animals

**Figure 2:**
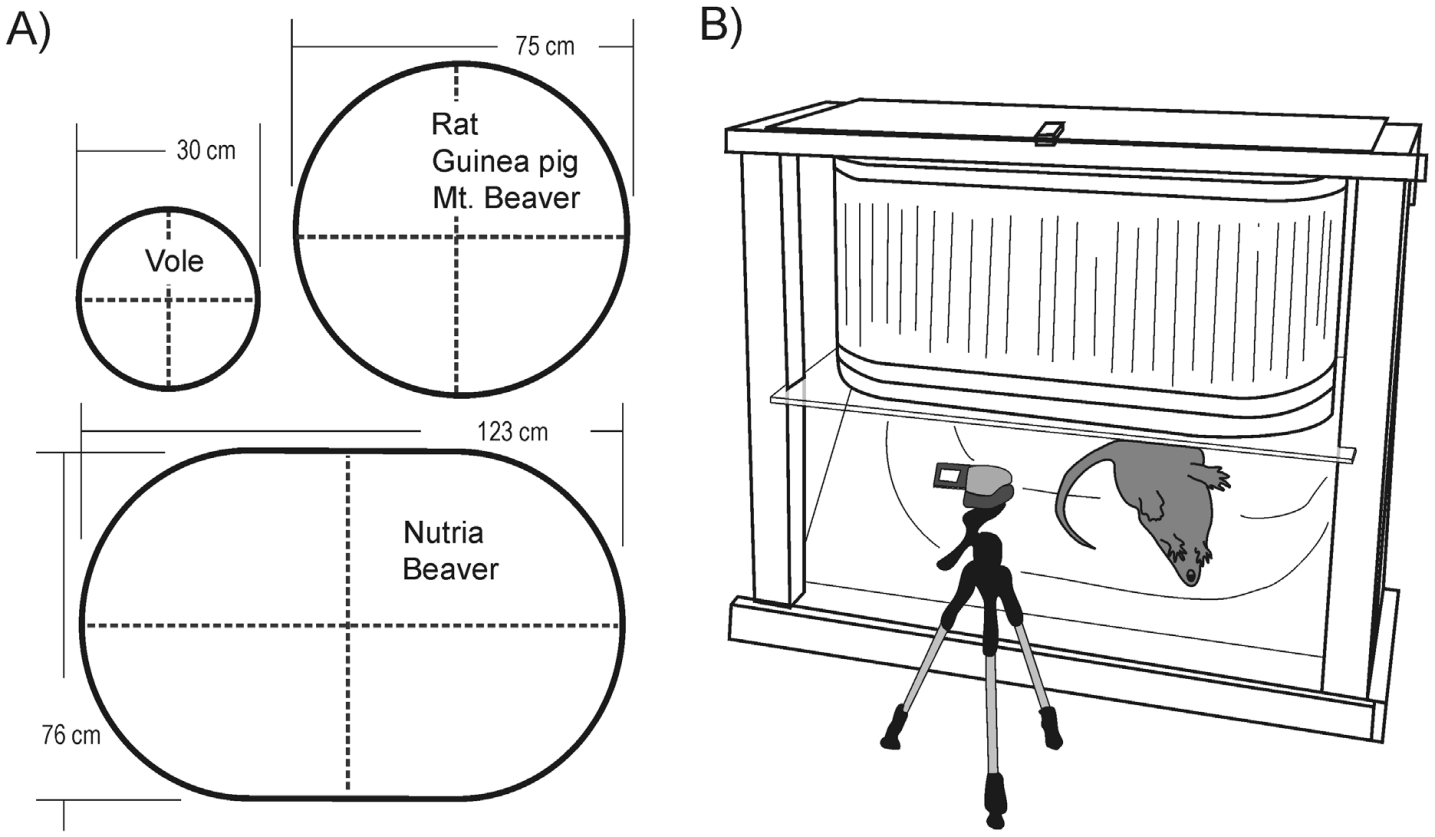
Behavioral test chambers. **A)** Floor surface areas for chambers used for behavioral testing in different rodent species. Dashed lines indicate the locations of quadrants used to score locomotion during video playback. **B)** Larger chamber used to test nutria and beaver. All test chambers had a clear glass floor and video recordings of the ventral surface of animals were collected by reflection in a mirror (45° angle). This design is based on taste reactivity testing, which is focused on the recording of mouth movements in laboratory rodents [80].

Test subject behaviors were recorded using digital video in specially constructed observation chambers (Fig. 2A). Circular chambers were used to test voles (30 cm in diameter and 60 cm tall) and rats, guinea pigs, and mountain beavers (75 cm in diameter and 75 cm tall) affixed to a clear Plexiglass plate serving as the floor of the chamber. Larger species (beaver and nutria) were tested in oval shaped chambers constructed with a 100 gallon stock tank placed on a 1.3 cm thick piece of clear glass (Fig. 2B). A mirror fixed at a 45° angle under the floor was used to facilitate observation and digital video recording (Sony DCR-SR300 or HDR-XR550V).

For most behavioral testing animals were injected with saline (sc or ig; 0.15 M NaCl) or emetic agents: copper sulfate (120 mg/kg, gavage, ig), veratrine (1 mg/kg, sc), or apomorphine (2 mg/kg). There were three exceptions to this dosing: 1) two guinea pigs received only 0.5 mg/kg apomorphine because reports show they are sensitive to this agent and it elicits gnawing behaviors [33] at this concentration (the other two received 2 mg/kg), 2) two guinea pigs were tested with 0.1 mg/kg veratrine and two were tested with 0.5 mg/kg veratrine, 3) rats received 5 mg/kg veratrine. All solutions were made in 0.15 M NaCl. Subcutaneous volumes were 2 ml/kg for voles and mountain beavers, 1 ml/kg for rats and guinea pigs, and 0.5 ml/kg for nutria and beavers; and, intragastric volumes, delivered by gavage needle (or flexible feeding tube in larger species), were 5 ml/kg for mice, rats, and mountain beavers, and 2 ml/kg for guinea pigs, nutria, and beavers. Larger species (mt. beaver, nutria, and beaver) were briefly anesthetized with 2–4% isoflurane via a face mask (Henry Schein) prior to insertion of the feeding tube for delivery of saline or copper sulfate solution. In these cases, animals were placed into the behavioral test chambers within 5 min after removal of the face mask. Each animal received no more than two tests of emesis, and tests were separated by at least 48 h. Animals were video-recorded for at least 40 min following an injection of an emetic agent or saline for subsequent analysis of behavior.

Digital video was scored offline using a computer monitor by two trained staff members blinded to the agent given. Parts of videos with behaviors indicative of emesis were reviewed by one author (CCH). Two types of observations were made: total movement and specific behaviors. To measure total movement, the observation chamber was split into four symmetrical quadrants on the video screen. When the animal moved from one quadrant to another, such that three of its four feet were within the new quadrant, the behavior was scored. Videos were carefully watched for the occurrence of any possible emetic movements (vomiting, retching, etc.) and other behaviors: cough (a brief forceful exhale), chewing nesting material, falling over, licking self, deep inhale (single expansion of the thorax), heaving (moving the head forward and mouth opening), digging, defecation, gnawing, grooming, head bobbing (moving the head back and forth), jumping, mouth movement, rolling over, overt salivation (drooling), shaking, scratching self, urination, and yawning/opening mouth. Percentages were calculated for each type of behavior based on the number of animals of the same species that displayed the behavior at any time during the observation period.

### Anatomical Measurements

**Figure 3:**
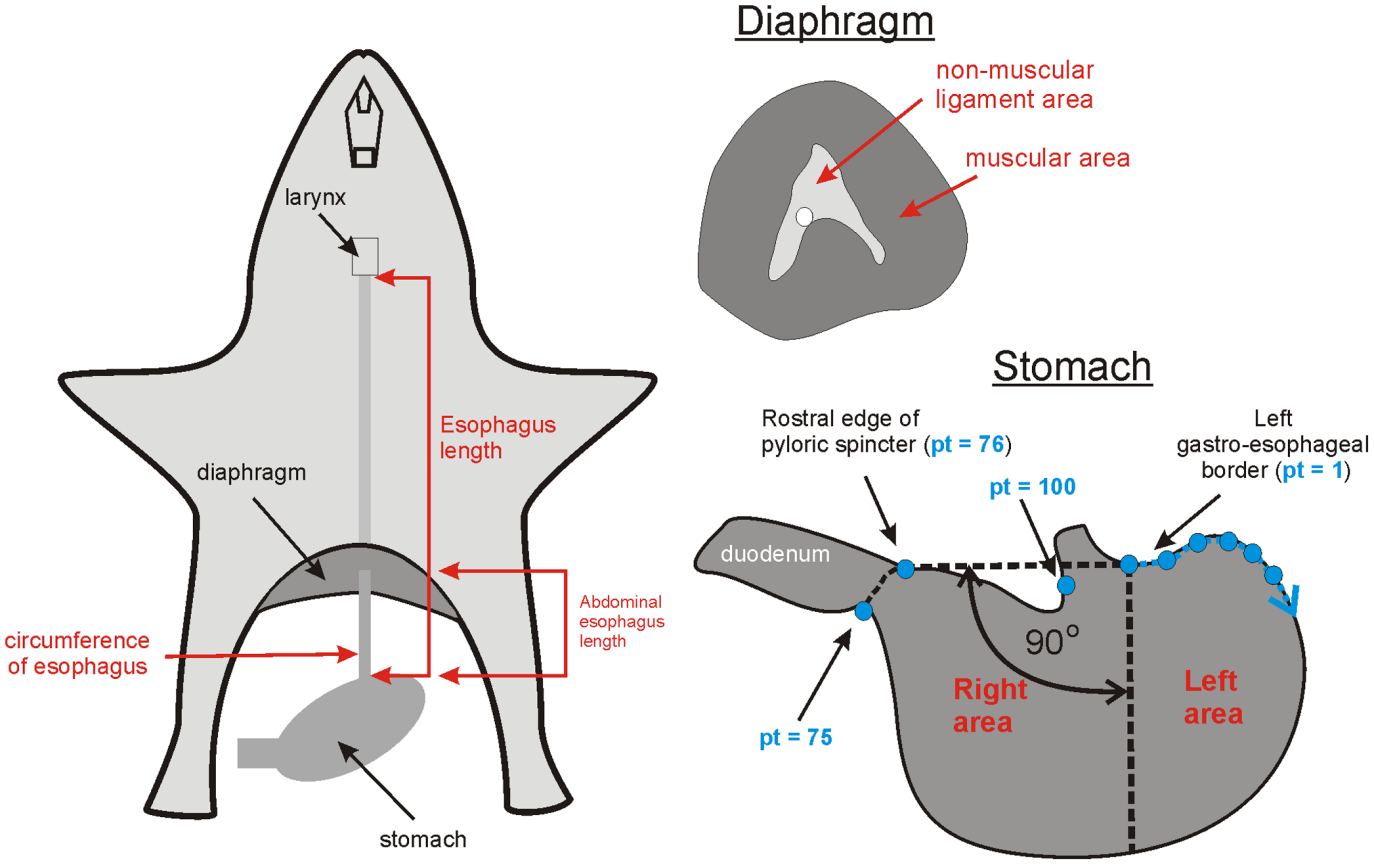
Anatomical measures of the esophagus, diaphragm, and stomach. Esophagus length measures were total (from gastroesophageal border to caudal extremity of larynx) and abdominal (below the diaphragm) components. Esophagus circumference was measured directly above the gastroesophageal border. The diaphragm was measured for muscular and non-muscular regions. Stomach shape was measured by placing a horizontal line on the gastroesophageal and gastroduodenal borders and creating a vertical division to determine left and right stomach surface areas. A measure of gastric shape included statistical analysis using 100 points, with 4 restricted landmark points (points 1, 75, 76, and 100) placed on the anatomical borders with the esophagus and duodenum (only a few of these points are shown in blue; starting at point 1 on the gastroesophageal border and moving clockwise).

A subset of animals used in behavioral testing was also used for anatomical measures of the esophagus, diaphragm, and stomach (Table 1). Animals were euthanized with CO_2_ exposure (mouse, vole, rat, guinea pig, mountain beaver, nutria, beaver, musk shrew), isoflurane exposure (4%) to achieve deep anesthesia followed by cardiac injection of Beuthanasia-D (Invervet/Merck; 1 ml, ferrets), or intravenous injection of sodium pentobarbital (120 mg/kg; cats). Esophageal measurements included the total and abdominal lengths, and circumference of abdominal segment (Fig. 3). Diaphragms and stomachs were carefully dissected *post mortem* and imaged flat on a table with back lighting. Esophageal measures were made by dissection of the neck to locate the larynx and the peritoneal cavity to isolate the stomach. Distance from the larynx to the diaphragm was measured externally by placing a thin metal rod at the diaphragm pointed vertically and measuring from this point to the caudal larynx. Distance from the diaphragm to the stomach was measured from the diaphragm to gastroesophageal junction (ventral surface) using a precision caliber or flexible tape measure. A cross-section of the abdominal esophagus was dissected and cut through the lumen and laid flat to measure circumference with a caliber. The diaphragm and stomach were dissected from the body cavity and placed on a lighted table and digitally imaged. All stomachs were partially filled and contents were kept in place for imaging. Calibrated area measures of the diaphragm and ventral stomach were collected using NIH ImageJ (http://rsb.info.nih.gov/ij/).

Stomach shapes were measured in two ways. First, to determine the relative position of the esophagus to the gastric compartment, a horizontal line was drawn from the left gastroesophageal border to the pyloric sphincter, which was used to determine the 90 degree vertical line separating the left and right stomach areas (Fig. 3). Second, ventral stomach images were processed using Corel Photo-Paint software to segment the contour and processed with the ImageJ plugin JFilament (http://athena.physics.lehigh.edu/jfilament/). The JFilament algorithm was used to detect two contours: equally spaced landmarks were used for the greater (75 points) and lesser (25 points) gastric curvatures (Fig. 3). Four points of the 100 points were anchored on the gastroesophageal and pyloric sphincter regions in order to align the stomachs for subsequent statistical shape analysis (Fig. 3) [34].

### Emetic Testing in an in situ Brainstem Preparation

**Figure 4:**
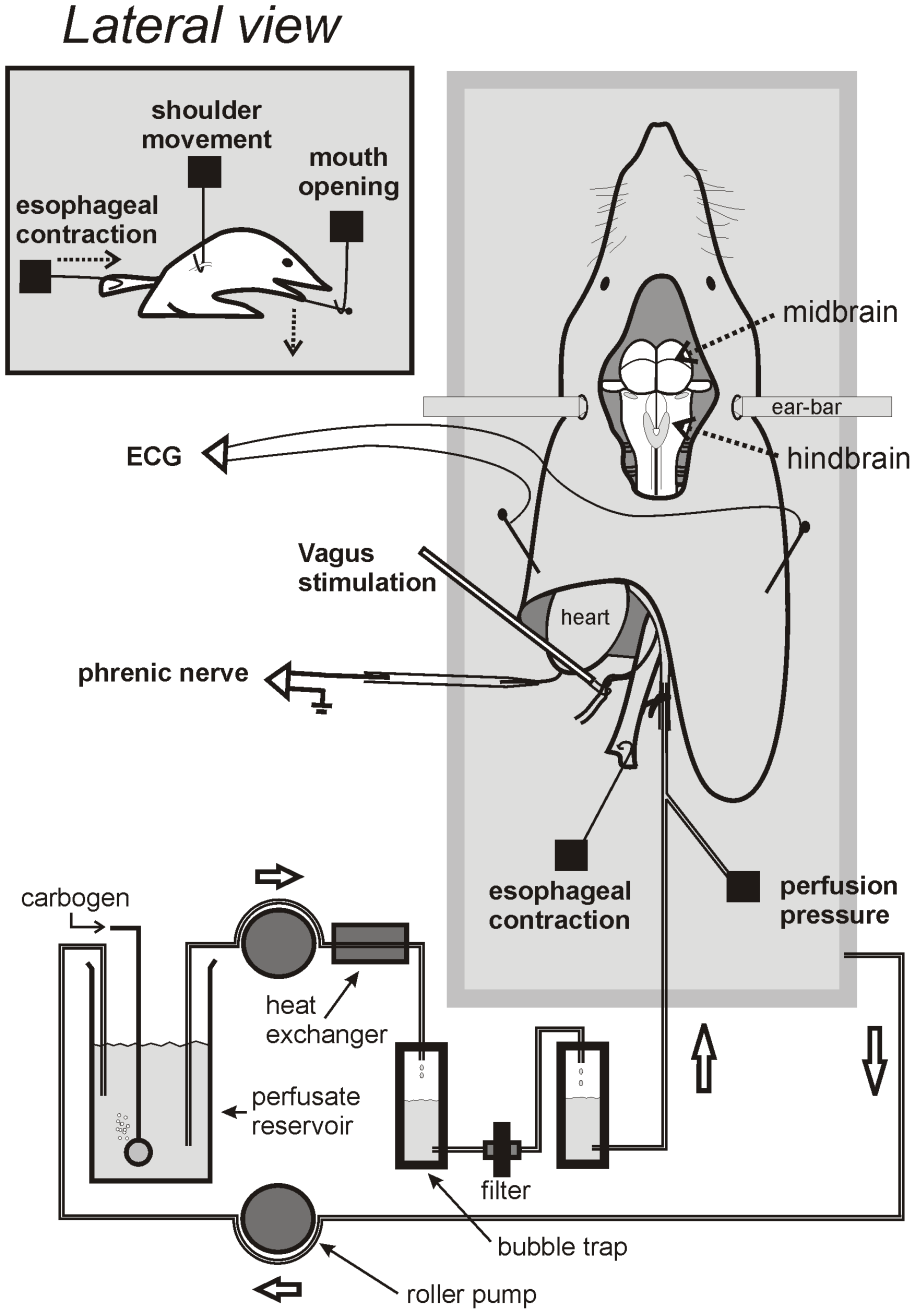
The *in situ* brainstem preparation for musk shrews, mice, and rats. Animals were deeply anesthetized, decerebrated, and perfused with artificial blood. Recordings included the phrenic nerve activity, esophagus and mouth contraction force, and shoulder displacement. Electrocardiogram (ECG) was recorded from pins placed in the lateral edges of the preparation. Perfusion pressure was measured with a pressure tranducer located close to tip of the aorta perfusion catheter. The location of vagus nerve electrical stimulation is also shown. This preparation is adapted from Paton and colleagues [28]–[30], [36].

The working heart brainstem preparation was used to conduct detailed recordings of mouth, esophagus, and shoulder movements, and neural activity of the phrenic nerve. The preparation was carried out as previously described for mice, juvenile rats, and musk shrews [28], [35] (Fig. 4). The juvenile rat was used in these experiments because rats in excess of approximately 100 g display inconsistent respiration cycles, due to the difficulty with maintaining sufficient perfusion pressure in a larger brain. In our experiments, we noted a similar problem in musk shrews. Male shrews (∼75 g) displayed inconsistent or no respiration patterns, therefore, females, which have a smaller brain and body size (∼40 g), were used.

Each animal was anesthetized with isoflurane (5%) until the pedal withdrawal reflex was absent. Animals were then transected below the diaphragm and placed into artificial cerebrospinal fluid (ACSF; 5–10°C) composed of the following chemicals: 1.25 mM MgSO_4_*7H_2_O, 1.25 mM KH_2_PO_4_, 3 mM KCl, 25 mM NaHCO_3_, 125 mM NaCl, 2.5 mM CaCl_2_*2H_2_O.

Following transection, the animal preparation was decerebrated above the superior colliculi. The preparation was placed on a Petri dish containing ACSF cooled over ice. The cerebellum and abdominal cavity organs were removed, keeping the esophagus to the level of the gastroesophageal junction. The diaphragm and part of the lungs were removed to isolate the phrenic nerve for recording. The descending aorta was isolated and the left ribs were removed to prepare for the catheter perfusion. The musk shrew had a larynx denervation in order to prevent sporadic apnea [28]. Lastly, a metal pin was placed between the teeth and the lower jaw, which was later connected to a force transducer to record mouth movements (Fig. 4).

Following the initial surgery, the preparation was placed into a custom-built perfusion chamber [36], and connected as shown in Figure 4. The head was secured with adjustable ear-bars and leveled. A 1.7 mm diameter double lumen catheter (Edwards Lifesciences) was placed into the aorta and perfusate was circulated. The circulating perfusate consisted of 250 ml of ACSF and 3.125 g Ficoll 70. A carbogen mixture of 95% O_2_/5% CO_2_ was bubbled through the perfusate using an airstone. Two bubble traps were connected to the system to prevent bubbles from damaging the brainstem. Perfusate temperature was maintained at 31–32°C using a heat pump (ThermoScientific P5). The perfusion rate was adjusted until the pressure was stable (Watson Marlow 520 s peristaltic pump). Then, the preparation was flushed with 50 ml of perfusate (to remove any remaining blood), resulting in a final amount of 200 ml of perfusate circulating during the experiment. Finally, vasopressin was added at a concentration of 400 pM in 10 µl in order to produce vasoconstriction and increase pressure. Sodium cyanide was added (10 µg/0.1 ml) in order to stabilize the respiratory rhythm [29].

Phrenic nerve efferent activity was recorded using a suction electrode connected to a high impedance headstage and amplifier (Grass Instruments, P511 AC). Amplification was set to 10–50 K, with band pass filtering of 100 Hz to 3 kHz. The amplified signal was then sent to a digital interface and computer (CED Power 1401 and Spike2 software; Cambridge Electronic Design) and recorded at 25 kHz. In preparations with stimulation of the vagal afferents, both vagal trunks were dissected from the esophagus and placed on two platinum-iridium hook electrodes attached to a stimulator (AM Systems). Mouth movements and longitudinal esophageal contraction were recorded using a force transducer (FORT25 and Transbridge amplifier; World Precision Instruments). A pressure transducer (DBP1000; Kent Scientific) was used to detect perfusion pressure and an electrocardiogram (ECG) was recorded by clips attached to metal pins inserted into the preparation. Force, pressure, and ECG measures were recorded with Spike2. Cardiorespiratory responses were then activated in order to establish the viability of the brainstem preparation. Infusion of 10–20 µg (0.1–0.2 ml) of sodium cyanide was used to activate peripheral chemoreceptors, which leads to a temporary reduction in the heart rate [29]. Perfusion pressure was maintained between 40 and 100 mmHg.

After stabilizing the preparation and establishing viability (heart rate, respiration), naloxone hydrochloride (Sigma-Aldrich) was added to the perfusate (80 µg in 100 µl; final perfusate concentration = 1 µM), as it is known to lower the emetic threshold in the *in situ* brainstem preparation of the musk shrew [28]. At least ten minutes later emesis was tested with either the addition of resiniferatoxin (RTX; Sigma-Aldrich) to the recycling perfusate (5 µg in 100 µl vehicle of Tween 80/ethanol/0.15 M saline, 1∶1∶8; final perfusate concentration = 40 nM) or the start of electrical stimulation. Electrical stimulation was applied with a silver bipolar hook electrode. Both vagal trunks were stimulated with pulses that were 0.2 ms wide and 30 Hz for 30 s duration for each voltage (isolated stimulator Model 2100; AM Systems). The initial stimulus voltage was 10 followed by 20, 5 and 2.5 volts, with at least a 4 min separation between stimulus conditions. RTX is an emetic agent in musk shrews, either free moving or in an *in situ* preparation [28], [37], and electrical vagal afferent stimulation produces emetic responses in musk shrews, ferrets, cats, and dogs [28], [38]–[40].

Offline detection of events in the recordings of the mouth, esophagus, shoulder and phrenic nerve was conducted using the threshold feature of DataView (http://www.st-andrews.ac.uk/~wjh/dataview/; University of St. Andrews, Dr. William Heitler). A 10 to 100 ms time filter was used to reduce the detection of very short events. Thresholds for the mouth, esophagus, and shoulder were set at a level that was slightly greater than events that occurred spontaneously (i.e., baseline) before application of RTX or electrical stimulation of the vagus. This strategy was used to capture only those events that were elicited by the putative emetic stimuli (after naloxone treatment). First responses after RTX were determined by finding the first esophagus or mouth movement (within 5 min from the start of emetic application), which exceeded the threshold and measuring the number of events for 15 s before and after this first event. If only an esophagus or mouth movement was detected, the same data were used in both the esophagus and mouth alignment analyses. If no esophagus or mouth events occurred, the average latency to the first event for each species group was used as the time of data collection for a given animal. For electrical stimulation, only the first 15 s of data after the start of the stimulus was used for analysis of esophagus, mouth, and shoulder movements. A 15 s sampling duration was selected because this can potentially capture several emetic episodes for a small animal with a rapid respiratory frequency [28] but is also a short period of time that will reduce the influence of other non-emetic related events. A tonic change in esophageal force was determined by measuring the average force during 5 s before and 5 s after the start of electrical stimulation (measurement regions were sometimes adjusted to less than 5 s to avoid any transient events).

### Data Analysis

**Figure 5:**
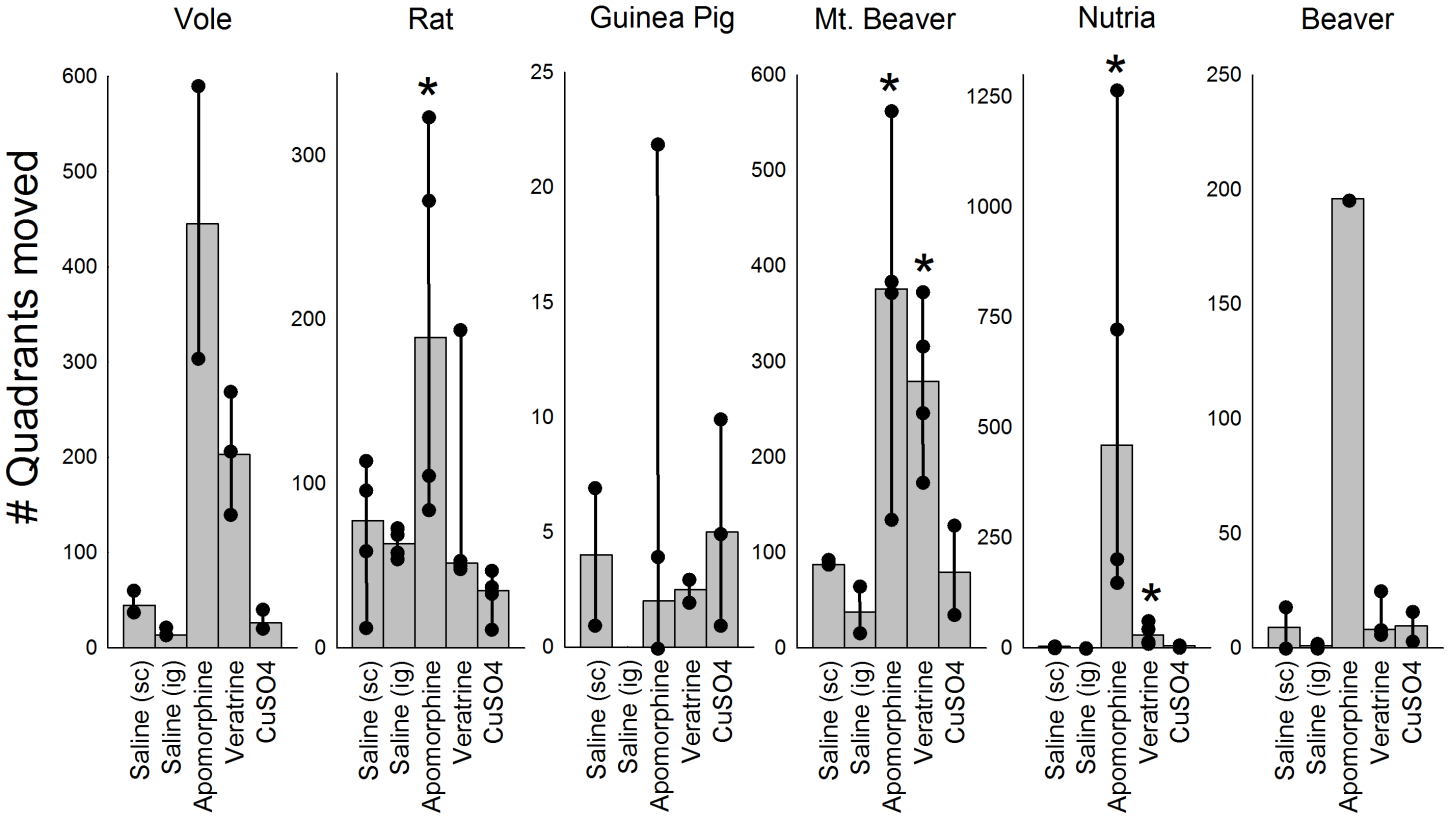
Effects of emetic agents on locomotion of rodent species. Vertical bars indicate median quadrants moved in the test chambers for each species group (see Fig. 2). Animals were injected with saline (sc or ig) or the emetic agents apomorphine (sc), veratrine (sc), or CuSO_4_ (ig) and observed for 40 min. Dark circles indicate raw movement scores for each animal and vertical lines represent the range of scores. * = p<0.05, Mann-Whitney U, comparison to saline control groups.

Behavioral movement data (quadrants; Fig. 5) were analyzed by Mann-Whitney U tests. Anatomical measures of the esophagus, diaphragm, and stomach were analyzed using one-way ANOVA and planned contrasts were used to compare the overall emetic group (musk shrew+ferret+cat) to each rodent species. Hotelling T^2^ statistic was used to compare the Rodentia and emetic groups for ventral stomach shape. In the *in situ* brainstem experiments, responses were analyzed separated for mouth, esophagus, or phrenic nerve burst counts (and at each voltage for electrical stimulation) across species using one-way ANOVA (or Kruskal-Wallis tests when a Shapiro-Wilk normality test failed, i.e., p>0.05). For RTX experiments, each species responses of the mouth, esophagus, or phrenic nerve counts were compared for 15 s before and 15 s after an event using paired Student t-tests (or Wilcoxon signed rank test when the normality test failed). For vagal electrical stimulation experiments, each species responses of the mouth movement counts, esophagus movement counts, phrenic burst counts, or esophageal force were compared across voltages using one-way ANOVA (or Kruskal-Wallis tests when a normality test failed). Comparison of group means was conducted using the Holm-Sidak method or Dunnett’s method (comparison to a single control group, 2.5 V). Statistical analysis was conducted using computer software (SigmaPlot, Systat; Statistica, Statsoft; or R, http://www.r-project.org/). Statistical significance was set at p<0.05.

## Results

### Emetic Testing in Free Moving Animals

In all species the amount of locomotion (number of quadrants moved) after copper sulfate (ig) was similar to saline control, which was relatively low throughout the 40 min observation period (Fig. 5). However, apomorphine produced a consistent increase in locomotion for rat, mountain beaver, and nutria (Fig. 5; p<0.05, Mann-Whitney U tests, apomorphine versus both sets of saline controls). Furthermore, mountain beavers showed an increase in locomotion after veratrine injection (Fig. 5; p<0.05, Mann-Whitney U test).

**Figure 6:**
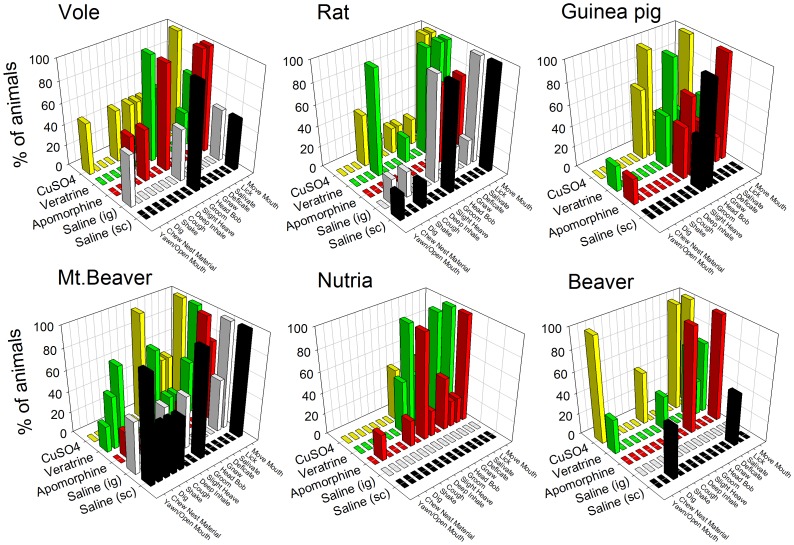
Behaviors scored after animals were injected with saline (sc or ig) or the emetic agents apomorphine (sc), veratrine (sc), or CuSO_4_ (ig). Data represent the percentage of animals of each species that showed specific behaviors for the 40 min test. No emetic responses were detected in any of these rodent species.

Although there were some pharmacological effects of the emetic agents on locomotion (Fig. 5), we observed neither retching nor vomiting responses in any of the rodent species. We plotted the 14 most commonly occurring behaviors in Figure 6, including mouth movements, licking, salivation, and grooming. In general, the emetic treatments produced more specific behaviors. Data are displayed as the percentage of animals showing each response during the 40 min period because the occurrence of each behavior was highly variable across animals and in most cases relatively low (1 to 5 occurrences).

### Anatomical Measurements

**Figure 7:**
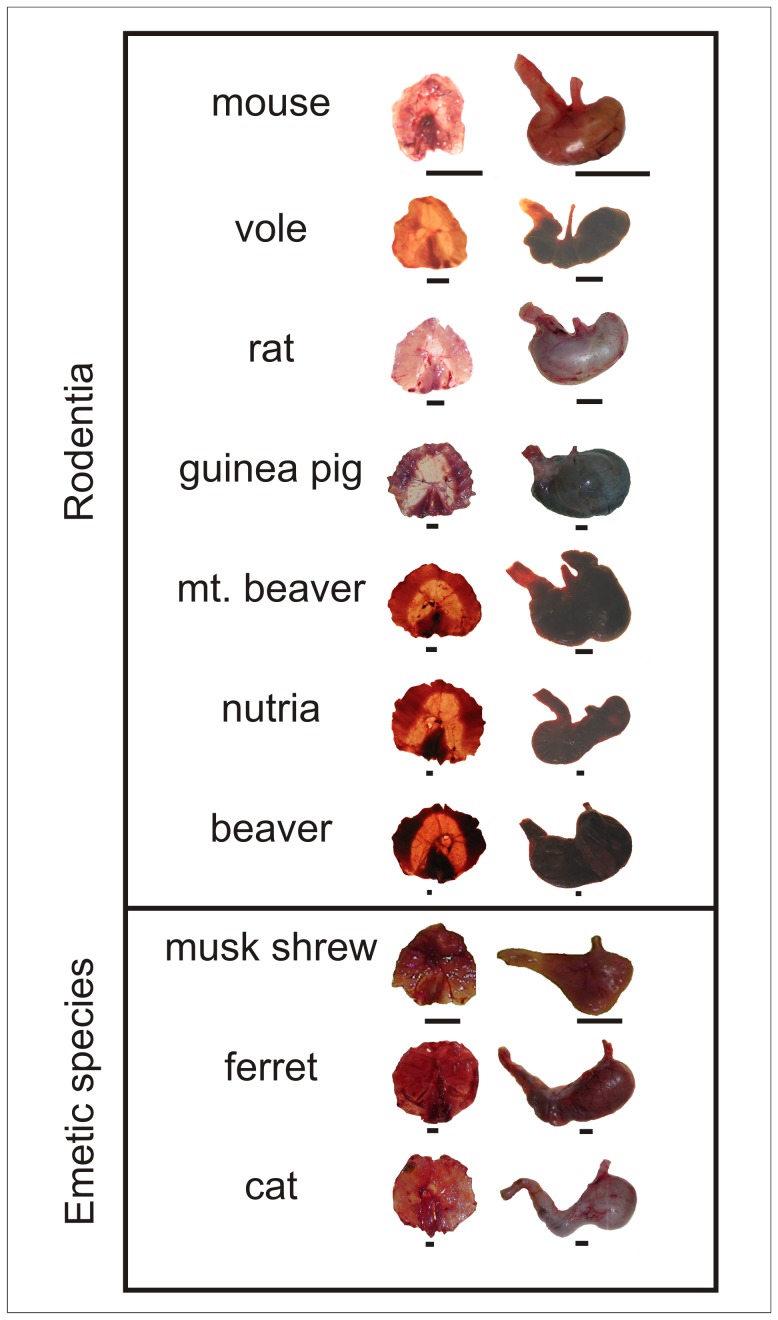
Representative anatomical images of the diaphragm and stomach in test species of Rodentia and emetic species. Bar = 1 cm.

**Figure 8:**
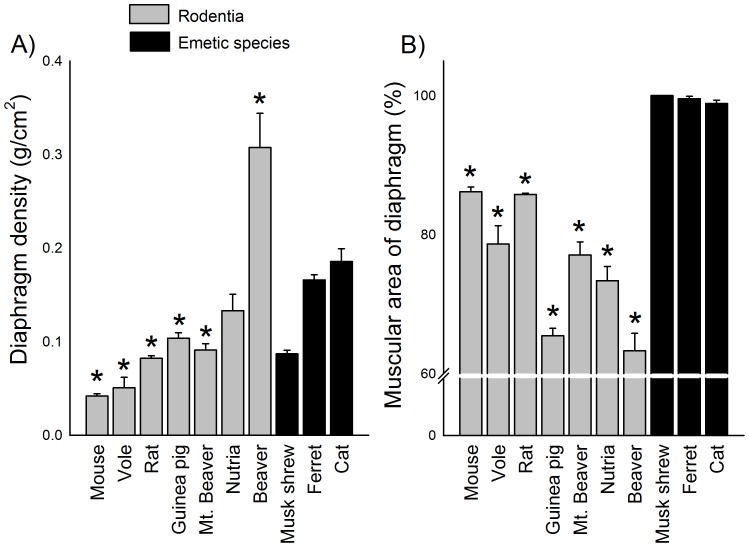
Diaphragm density and area measures. **A)** Density of the diaphragm (g/cm^2^). **B)** Percentage of diaphragm area composed of muscle compared to ligament. The SEM for musk shrews is small and hidden by the vertical bar. See Figure 3 for a diagram showing the location of these measures. * = p<0.05, planned contrast, a rodent species compared to all emetic species. Data represent mean ± SEM.

Figure 7 shows representative anatomical specimens for the diaphragm and esophagus of rodents and emetic species. The diaphragm had a lower density in mouse, vole, rat, guinea pig, and mountain beaver compared to the emetic group [F(9,48) = 38.7, p<0.05, one-way ANOVA; Fig. 8A, p<0.05, planned contrasts]. However, beavers displayed a higher density compared to the emetic group (Fig. 8A; p<0.05, planned contrast). All rodent test species showed a smaller muscular area and a larger central tendon in the diaphragm compared to the emetic group [F(9,48) = 149.4, p<0.05, one-way ANOVA; Fig. 8B, p<0.05, planned contrasts].

**Figure 9:**
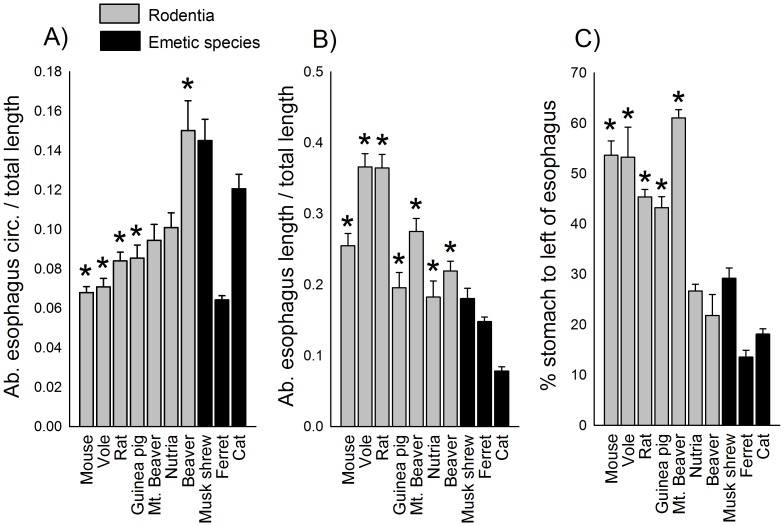
Esophagus and stomach area measures. **A)** Abdominal esophagus circumference/total esophagus length (cm). **B)** Abdominal esophagus length/total esophagus length. **C)** Percentage of stomach area to the left of vertical division. See Figure 3 for a diagram showing location of these measures. * = p<0.05, planned contrast, a rodent species compared to all emetic species. Data represent mean ± SEM.

The ratio of the abdominal esophagus circumference to total length was smaller in mouse, vole, rat, guinea pig, and mountain beaver compared to the emetic group [F(9,48) = 16.6, p<0.05, one-way ANOVA; Fig. 9A, p<0.05, planned contrasts]. However, the ratio of the abdominal esophagus length to total esophagus length was greater for all rodents compared to the emetic group [F(9,48) = 32.4, p<0.05, one-way ANOVA; Fig. 9B, p<0.05, planned contrasts]. Mouse, vole, rat, guinea pig, and mountain beaver had a larger extension of the proximal stomach region (% of the stomach to the left of the gastroesophageal border) compared to the emetic group [F(9,47) = 45.3, p<0.05, one-way ANOVA; Fig. 9C, p<0.05, planned contrasts], which indicates a more medial position for the esophagus position on the stomach.

**Figure 10:**
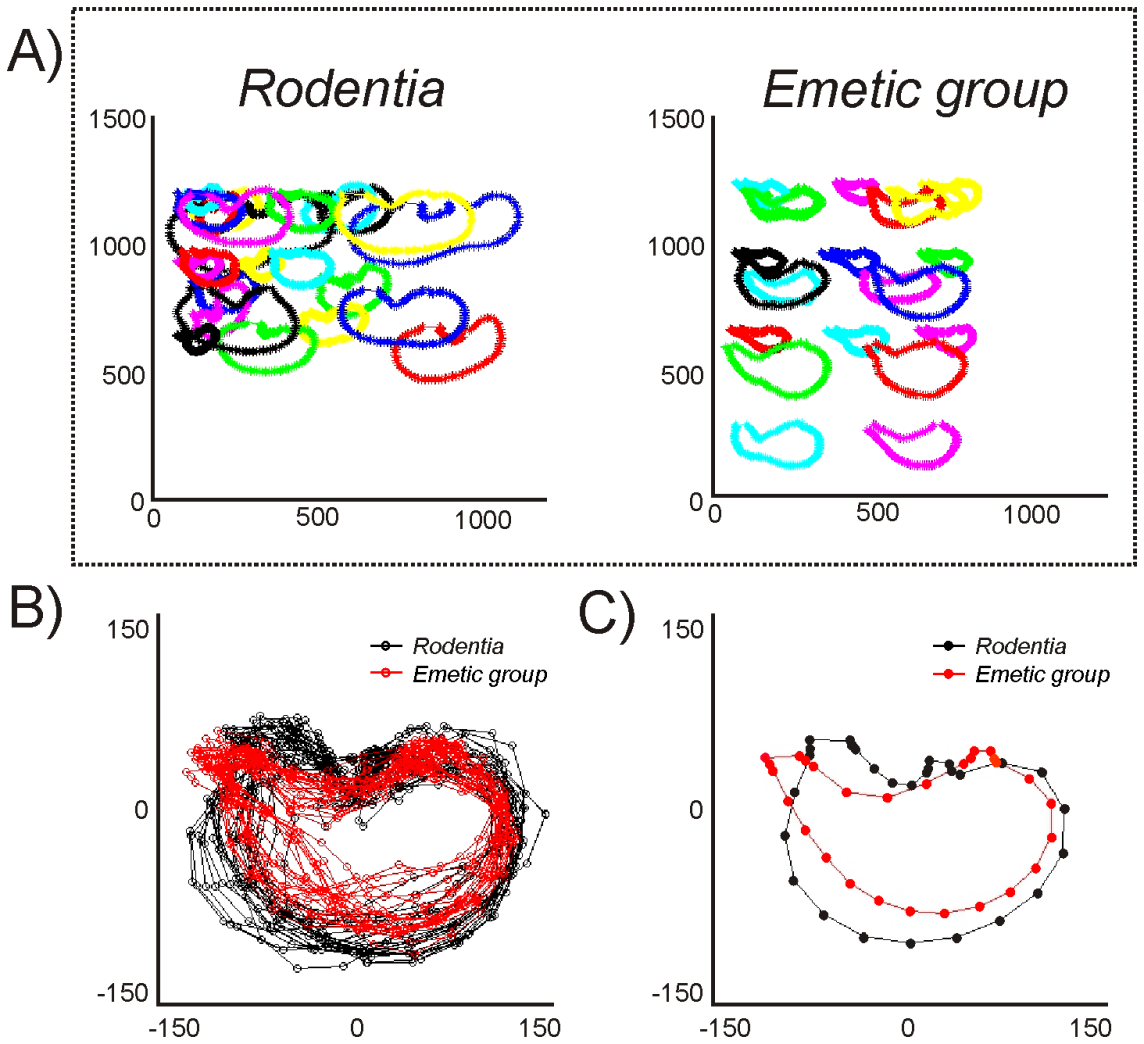
Stomach shape analysis. **A)** Initial X and Y coordinates of ventral stomach shapes for the Rodentia group (n = 32) and emetic group (n = 25). **B)** All stomach shapes were aligned using a Procrustes analysis (including translation, rotation, and scaling). **C)** Average group values for the Procrustes transformed stomach shapes: Rodentia (in black) and emetic group (in red). Hotelling T^2^ statistic = 0.59, p<0.001, Rodentia compared to emetic group. X and Y coordinates in each figure represent arbitrary units in image graphics.

A statistical analysis of the stomach shapes indicated that Rodentia is different from the emetic group (Hotelling T^2^, p = 0.001; Fig. 10C). The analytical steps are shown in Figure 10. Figure 10A shows the raw X and Y coordinates for stomach shapes for the Rodentia and emetic groups. Four mice were randomly selected from the original 8 to reduce the influence of mouse on the statistical shape analysis. The final analysis included 28 rodents (4 mice, 4 voles, 4 rats, 4 guinea pigs, 4 mountain beavers, 4 nutrias, and 4 beavers) and 25 emetic animals (9 shrews, 8 ferrets and 8 cats). Twenty-eight landmarks from the original 100 (Fig. 3) were selected to increase the weighting of those points that were *a priori* landmarks (i.e., points where the esophagus and intestine attach to the stomach). Points included in the analysis were numbers 1, 2, 3, 8, 13, 18, 23, 28, 33, 38, 43, 48, 53, 58, 63, 68, 73, 74, 75, 76, 77, 78, 83, 88, 93, 98, 99, and 100 (Fig. 3). Data were pooled for all animals and a general Procrustes analysis (including translation, rotation and scaling) was performed in the shapes package in R software (http://www.r-project.org/; Fig. 10B). A mean shape between the Rodentia and emetic groups was compared using tangent coordinates (where the overall average shape was used as the reference shape; Fig. 10C) and Hotelling T^2^ statistic [41]. P-values are based on resampling and a permutation test. Permutation resampling was carried out without replacement in pooled samples which had been transformed with general Procrustes analysis. The “testmeanshapes()” function in the shapes package of R software was used. The number of permutations was 1000. The Hotelling T^2^ statistic was 0.61, p = 0.001.

### Emetic Testing in an in situ Brainstem Preparation

Heart rate and phrenic nerve measures were assessed for 30 s before naloxone and 30 s before RTX or electrical stimulation of the vagal afferents (i.e., 10 min after naloxone). Addition of naloxone to the brainstem perfusate did not affect heart rate in mice (before, 479±69 and after, 544±53, beats/min; p>0.05, t-test) or rats (before, 338±14 and after, 376±12, beats/min; p>0.05, t-test). Naloxone produced an increase in heart rate in musk shrews (before, 216±23 and after, 259±27, beats/min; t(13) = 3.7, p<0.005] but there was no statistical difference between the three species (difference scores, before minus after; p>0.05, Kruskal-Wallis one-way ANOVA). In contrast, there was a statistically significant change in phrenic nerve bursting across species after naloxone (difference scores; p<0.05, Kruskal-Wallis one-way ANOVA), and musk shrews displayed an increase in bursting (before, 18±2 and after, 49±12, bursts/min; p<0.05, Wilcoxon rank test) compared to mice (before, 33±6 and after, 31±4, bursts/min; p>0.05, Wilcoxon rank test) and rats (before, 15±0.8 and after, 17±2, bursts/min; p>0.05, Wilcoxon rank test).

**Figure 11:**
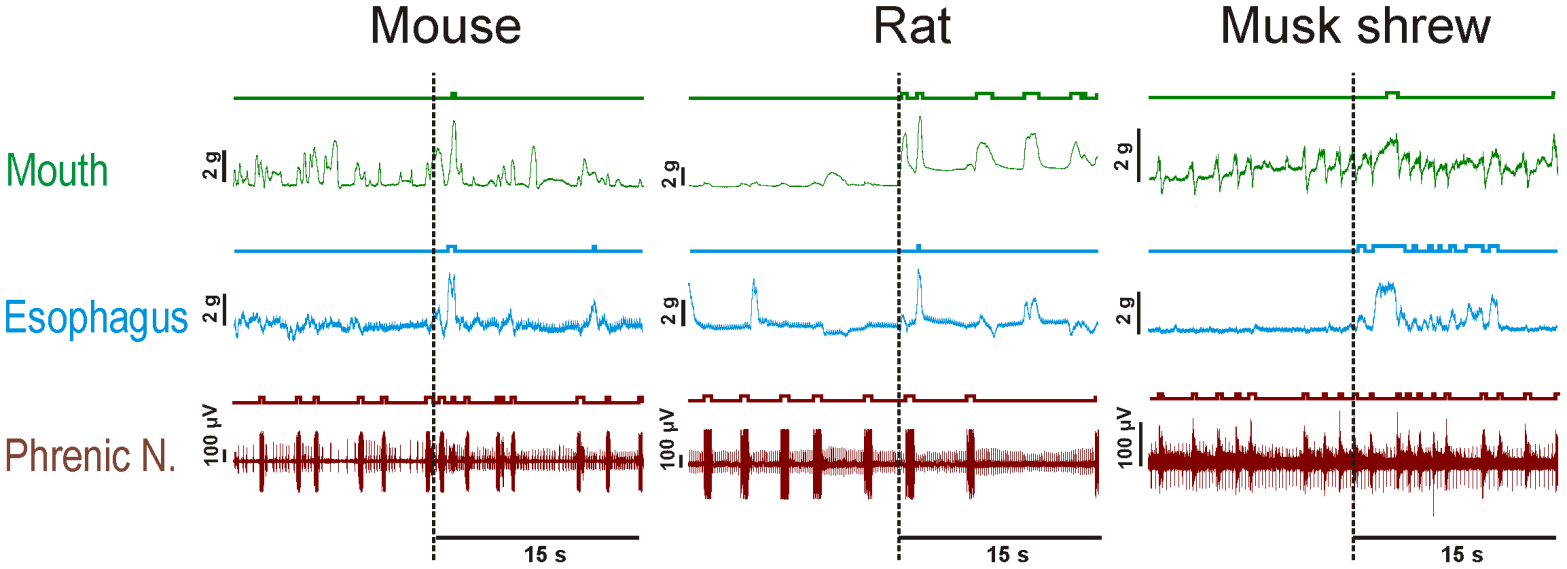
Representative recordings of the mouth movement, esophagus movement, and phrenic nerve activity from the mouse (C57BL6), rat (Sprague-Dawley), and musk shrew in the *in situ* brainstem preparation. Vertical dashed lines indicate the start of the contraction of the esophagus after resiniferatoxin (RTX) was perfused through the brainstem (Fig. 4). Plots show 15 s pre-event versus 15 s post-event (see Fig. 12 for group averages). Mouth and esophageal recordings indicate force (g), with positive deflections showing opening of the mouth and shortening of the esophagus. Lines and event marks above each trace indicate events detected by computer software (DataView; http://www.st-andrews.ac.uk/~wjh/dataview/).

**Figure 12:**
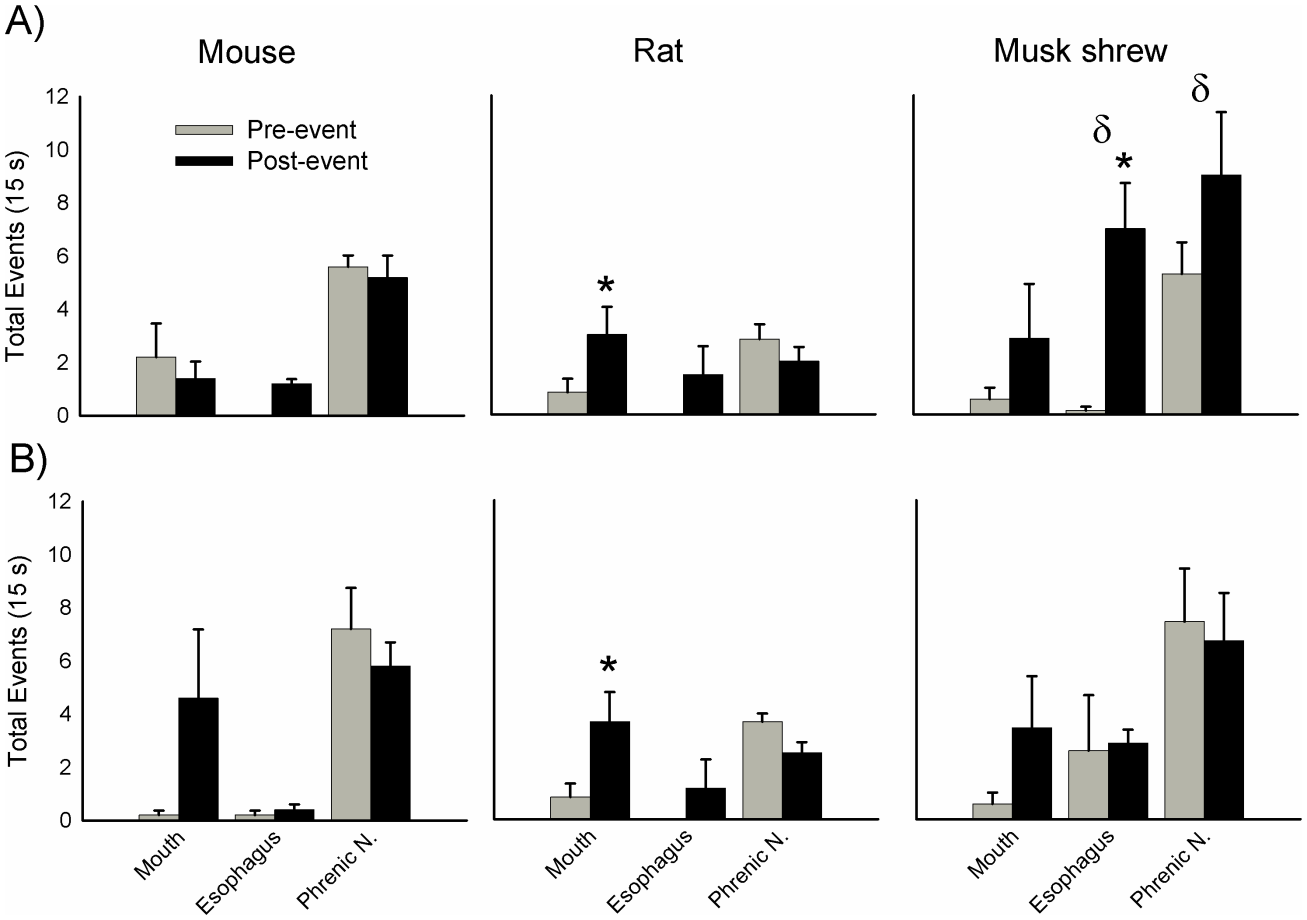
Average effects of resiniferatoxin (RTX; 40 nM) treatment on mouth, esophagus, and phrenic nerve responses from the brainstems of mouse (C57BL6), rat (Sprague-Dawley), and musk shrew (Fig. 4). **A)** Mouth, esophagus, phrenic nerve events during the 15 s before and after (pre- and post-event) alignment to the first large esophageal movement (an esophageal movement that was greater than baseline movements). **B)** Effects when data are aligned to the first large mouth movement. * = p<0.05, Wilcoxon Signed Rank test, number of pre-events versus number of post-events. δ = p<0.05, Kruskal-Wallis one-way ANOVA, species effect for difference between pre-and post-event values. Data represent mean ± SEM.

After RTX injection, shrews showed the first indication/sign of an emetic-like event at a median of 67 s, based on the movement of the esophagus, and at 78 s based on the movement of the mouth. Corresponding latencies were 224 s and 122 s for mice and 90 s and 60 s for rats. Representative recordings of the mouth, esophagus, and phrenic nerve responses are shown in Figure 11. Rats displayed statistically significant differences between pre and post number of mouth events when data were aligned by esophagus or mouth movements (Fig. 12A and 12B; p<0.05, Wilcoxon sign rank tests). When data were aligned by the first esophagus movement, shrews displayed a statistically significant increase in esophagus movements (Fig. 12A; p<0.05, Wilcoxon sign rank test). There were statistically significant species effects for the esophagus and phrenic nerve responses, but only when data were aligned to the first esophagus movement (Fig. 12A; p<0.05, Kruskal Wallis one-way ANOVA using the pre- and post-event difference scores; p<0.05, Dunn’s comparison between shrew and rat).

**Figure 13:**
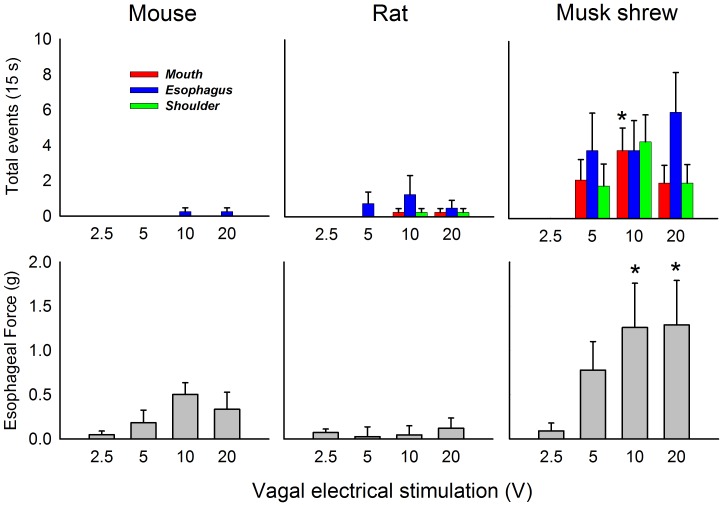
Average effects of vagal afferent electrical stimulation (2.5, 5, 10, and 20 V applied for 30 s) on mouth, esophagus, and phrenic nerve responses from the brainstems of mouse (C57BL6), rat (Sprague-Dawley), and musk shrew (Fig. 4). *Top row:* The effects of stimulation on mouth, esophagus, and shoulder movements. ***Bottom row:*** The effects of stimulation on tonic esophageal force (measured in the first 5 s after the start of stimulation). * = p<0.05, Dunnett’s test, versus 2.5 V condition. Data represent mean ± SEM.

Electrical stimulation of the vagal afferents, from 5 to 20 V, produced consistently large coordinated responses in mouth, esophagus, and shoulder movements in musk shrews but not rats or mice (Fig. 13, top). Moreover, only shrews showed a statistically significant increase in mouth movements across the range of voltage, and 10 V produced significantly greater mouth movements compared to 2.5 V [F(3,15) = 3.6, p<0.05; one-way repeated measures ANOVA; p<0.05, Holm-Sidak test, mean comparison]. Furthermore, only shrews displayed statistically significant increases in esophageal force across the voltage range and 10 and 20 V produced a significantly greater force than 2.5 V [Fig. 13, bottom; F(3,15) = 3.5, p<0.05, one-way repeated measures ANOVA; p<0.05, Dunnett’s test, mean comparison to 2.5 V]. Both RTX and vagal electrical stimulation produced mouth, esophagus, shoulder, and phrenic nerve bursting in the *in situ* preparation of the musk shrew that are consistent with emetic-like episodes [28].

## Discussion

None of the rodents tested in free moving behavioral assays showed either retching or vomiting after injection of drugs known to induce emesis in the dose range and observation time used in multiple species known to be capable of emesis [9], [23], [42]–[48]. In addition, there were notable differences in diaphragmatic structure (relatively less muscular), intra-abdominal esophagus length (relatively longer in non-emetic species), and stomach geometry (absence of a funnel shape) that might limit the ability of rodents to vomit. Detailed *in situ* brainstem testing of laboratory rats and mice indicated that these animals do not have the diagnostic signs of the coordinated components of an emetic-like episode seen in the musk shrew, i.e., multiple closely spaced longitudinal esophageal contractions and phrenic nerve bursting activity (fictive emesis) with an associated mouth opening [28].

The selected rodent species did not retch or vomit after administration of the prototypical emetic agents apomorphine (sc), veratrine (sc), or copper sulfate (ig). There is little doubt that these three emetic agents produce both retching and vomiting within 15 min in emetic species. Apomorphine (up to 2 mg/kg, sc) produces emesis in ferrets, dogs, mink, and least shrews with a latency of 2 to 10 min (e.g., [23], [42]–[44]). Veratrine (up to 1 mg/kg, sc) induces emesis in musk shrews with a latency of approximately 7 min (e.g., [9], [45]). Copper sulfate (up to 120 mg/kg, po) produces emesis in dogs, ferrets, and musk shrews with a latency of 2 to 15 min (e.g., [46]–[48]). It is unlikely that emetic events were missed in the current analyses because many subtle behaviors, such as respiratory and oral movements, that are components of emetic responses were recorded. In the few cases where these rodents showed a cough or slight heave, these events were quite different from the sequence of retches that accompany vomiting in a species with an emetic response. For example, a sequence of retches most often leads to a vomit (multiple retches and a vomit together form an emetic episode) but single retches or vomits are sometimes reported as isolated events in musk shrews and ferrets [27], [49], [50]. Retches and vomits involve a different sequence of muscle contractions [51], [52]: 1) Retches are produced by synchronized contraction of the crural and costal diaphragm and abdominal muscles resulting in a net increase in intra-abdominal pressure (to position the contents of the stomach for expulsion [49]), and 2) Vomits are composed of contraction of the costal diaphragm, abdominal, and intercostal muscles resulting in a net increase in both intra-abdominal and intra-thoracic pressures (to eject the gastric contents out of the mouth). However, the current emetic treatments did produce overt salivation (drooling) in these rodents. Salivation is sometimes associated with nausea and emesis in humans [53] and is reported prior to the onset of retching and vomiting in laboratory animals with an emetic reflex (e.g. cat, dog [38], [54]). It is evident that apomorphine, and the other two chemical agents, had an impact on animal behavior at the selected doses (Fig. 5 and 6). For example, apomorphine produced a consistent increase in locomotion, a known effect of D_2_ receptor agonists [55], [56].

It appears that individually none of the anatomical features that were measured explains the lack of emesis. In contrast, some of these anatomical metrics might indicate that it would be difficult for rodents to efficiently vomit. For example, rodents have reduced diaphragmatic muscle to assist in abdominal pressure changes [57]. Uniquely, although the beaver had a high level of diaphragm density with heavy muscularity in edges of the costal (lateral) and crural (central) diaphragm areas, it had a large central tendon region devoid of muscle (Fig. 7 and 8). A previous study indicated that non-vomiting species have a longer and narrower abdominal esophagus relative to overall esophagus length compared to species that vomit [14], but we were unable to reproduce this finding (Fig. 9A and 9B). The ratio of abdominal esophagus circumference to total length in ferrets was notably similar to several rodents (Fig. 9A). Although the rodents displayed a significant difference in abdominal esophagus length to the total length compared to the emetic group, musk shrews and ferrets were close to the values measured in guinea pig and nutria (Fig. 9B). The percentage of the stomach to the left of the esophagus also did not completely distinguish rodents from the emetic group (Fig. 9C), which suggests that the position of the esophagus alone might not be a distinguishing feature. However, a statistical shape analysis indicates that stomach shape could be an important feature of emetically competent animals. The emetic group showed a more funnel-shaped stomach compared to the rodent group (Fig. 10) and this geometry could facilitate the movement of gastric contents into the esophagus.

Free moving behavioral testing cannot address the possibility that rodents have more subtle emetic responses, perhaps existing as a degenerate reflex. This is the power of the *in situ* brainstem approach. With this methodology we were able to make sensitive measures of the force of the esophagus and mouth movements, shoulder displacement, and phrenic nerve bursting activity; all of these features are critical components of emetic responses [28], [52]. It is clear from these sensitive measures that laboratory mice (C57BL6) and rats (Sprague-Dawley) do not display the coordinated actions associated with an emetic reflex produced by two standard emetic stimuli – RTX treatment and vagal afferent electrical stimulation [28] (Fig. 12 and 13). Our data also indicate that rodents have an inability to longitudinally shorten the esophagus after vagal afferent electrical stimulation (Fig.13, bottom), which was previously reported in an *in vitro* study of the mouse and musk shrew esophagi [58]. RTX produces emesis by a centrally-mediated mechanism involving the stimulated release of substance P in the caudal hindbrain [37]. The action of substance P on NK_1_ receptors in the emetic circuitry is strongly supported across several emetic models, including musk shrews, ferrets, cats, and dogs [51]. Vagal afferent electrical stimulation is also a well established prototypical test stimulus to drive emetic responses in *in vivo* physiology experiments using musk shrews, ferrets, cats, and dogs [28], [39], [59], [60].

Although rodents have peripheral musculature and gastrointestinal physiology (e.g., absence of functional motilin [17]) that is dissimilar from humans and other animals with a vomiting reflex, the current report also suggests differences in CNS circuitry that could explain the lack of emesis in rodents. Several metrics of emesis in the present report, including mouth, shoulder, and phrenic nerve responses do not involve the gastrointestinal musculature and still did not display an emetic-like pattern in mice and rats. The results point to the probability that rodents lack critical brainstem emetic circuitry that can generate patterned emetic responses. Indeed evidence from neuronal tracing studies indicates that ferrets and cats have a large number of medullary midline neurons that provide input to phrenic motor neurons [61], [62], which is not observed in the rat [63]. Anatomical tracing studies indicate that these midline neurons are also possible integrators of both diaphragmatic and abdominal responses [64]. Despite the lack of emesis in rodents, it can be argued that rodents (rat, mouse), and emetic species (ferret, musk shrew) both experience “nausea” or visceral sickness, which is indicated by conditioned taste aversions that are produced by emetic stimuli [65]–[68]. Moreover, nauseogenic stimuli, such as illusory self-motion and cholecystokinin injection, produce a large increase in neurohypophyseal secretion of vasopressin but little to no secretion of oxytocin in humans who report nausea [69], [70]. Similarly, emetic species [71]–[73] show a rise in vasopressin after injection of emetic agents but rats show an opposite response – elevated oxytocin and little to no vasopressin release [74], [75]. This suggests that there are differences in nauseogenic activation between rodents and emetic species that extend beyond the caudal hindbrain.

The current study is limited in scope to those rodents that were included. Rodents tested in the current report are a small selection of the approximately 1800 species of rodents [12]. However, it is clearly neither practical nor ethical to conduct large scale behavioral testing of Rodentia. Importantly, the current study represents the first experimental test for emetic responses using species from the three major divisions of Rodentia (Fig. 1). The emetic species used for comparison are obvious choices because musk shrews, ferrets, and cats are standard emetic models [13]. The one outlier is the lack of dog anatomical samples. It does not appear that the addition of dog measures would add much to the anatomical analysis since for several of the measures there was significant intra-group variability in the emetic species that exceeds the variability measured by comparison to the selected rodents.

In summary, the current study shows that the inability to vomit is likely a general phenotype of rodent species. The current report represents the first detailed experimental study of the lack of emesis in diverse rodents. The strengths of this study include the combined use of free moving behavioral testing, detailed anatomical measures, and use of an *in situ* brainstem physiology preparation to collect sensitive measures of mouth, esophagus, and shoulder movements, and phrenic nerve bursting activity. These findings indicate the need for CNS neurophysiological studies to directly compare rodents and emetic species following activation of possible nausea-related pathways and the absence or presence of the critical motor output pathways of the emetic reflex. Understanding the lack of emesis in rodents has implications for the suitability of typical laboratory species, such as rats and mice, for the study of nausea and vomiting (Chap. 8 in [16]; [17]).
