# Correction: Why Can’t Rodents Vomit? A Comparative Behavioral, Anatomical, and Physiological Study

**DOI:** 10.1371/annotation/1c75cd5d-9dde-4ace-8524-a4980745e804

**Published:** 2013-06-21

**Authors:** Charles C. Horn, Bruce A. Kimball, Hong Wang, James Kaus, Samuel Dienel, Allysa Nagy, Gordon R. Gathright, Bill J. Yates, Paul L. R. Andrews

The first author's affiliations were listed incorrectly. Charles C. Horn is not only affiliated with institutions 1 and 4, but also with institution 2, Department of Medicine: Division of Gastroenterology, Hepatology, and Nutrition, University of Pittsburgh School of Medicine, Pittsburgh, Pennsylvania, United States of America, and institution 3, Department of Anesthesiology, University of Pittsburgh School of Medicine, Pittsburgh, Pennsylvania, United States of America. 

